# Surgery of the Brain

**Published:** 1888-10

**Authors:** 


### EDITORIAL.

### SURGERY OF THE BRAIN.

With the long-established operation of trephining in cases of injury to the brain, and the recent advances in thoracic and abdominal surgery, it seemed as if the fields open to surgery had been about all cultivated, but with the report of Macewen, made at the last meeting of the British Medical Association in Glasgow, in August, and the more recent report in the Congress of Physicians and Surgeons in Washington, last month, of the work of Mills and Weir and Horsley and others, it seems established that it is no longer necessary to stop with trephining the skull; that the brain itself may now be invaded in some cases not only with safety, but with surprising advantage. Whether the lovers of notoriety will be as reckless hereafter in brain surgery as they seem to have become in abdominal surgery remains to be seen. With some, evident desire for notoriety seems so irresistible that in all probability the near future must witness the passage beyond a line of safety into the wide domain of uncertainty and risk, and sometimes to the detriment of the patient.

If laparotomy, to verify diagnosis, be justifiable, will not some reckless or injudicious diagnostician want to verify a diagnosis of compression of the brain, and feel more free to trephine the skull, only to find that his supposed case of compression proved to be but the stupefaction produced by alcohol? It seems to be as difficult as ever to avoid excessive conservatism on the one hand and reckless surgery on the other. Now that prudent surgeons have shown that surgery may advance somewhat beyond what were supposed to be its limits of safety, the doors have been opened for the imprudent ones. How soon shall the reports of the first victims be looked for? Scylla may be avoided, but let not Charybdis claim the escaped as victims.

